# Who are diabetic foot patients? A descriptive study on 873 patients

**DOI:** 10.1186/2251-6581-12-36

**Published:** 2013-07-05

**Authors:** Nima Madanchi, Ozra Tabatabaei-Malazy, Mohammad Pajouhi, Ramin Heshmat, Bagher Larijani, Mohammad-Reza Mohajeri-Tehrani

**Affiliations:** 1 Endocrinology and Metabolism Research Centre, Tehran University of Medical Sciences, Tehran, Iran; 2 Endocrinology and Metabolism Research Centre, Tehran University of Medical Sciences, Shariati Hospital, North Kargar Street, 14114, Tehran, Iran

**Keywords:** Diabetes mellitus, Diabetic foot, Ulcer, Patient care

## Abstract

**Background:**

Diabetic foot ulcer (DFU) as the leading cause of lower limb amputation is one of the most important complications of diabetes mellitus (DM). Patient and physician’s education plays a significant role in DFU prevention. While effective treatment and formulation of prevention guidelines for DFU require a thorough understanding of characteristics of DFU patients and their ulcers, there are reports that not only patients’ but also physicians’ information about these characteristics is inadequate. So we conducted this study to investigate these characteristics.

**Methods:**

Necessary data was collected from medical archives of DFU patients admitted between 2002 and 2008 in two university hospitals.

**Results:**

873 patients were included. Mean age was 59.3 ± 11.2 years and most of the patients developed DFU in 5th and 6th decades of their life. 58.1% were men. 28.8% had family history of DM. Mean duration of DM was 172.2 months. Mean duration of DFU was 79.8 days. Only 14.4% of the patients had Hemoglobin A1C < 7%. 69.6% of the patients had history of previous hospitalization due to DM complications. The most prevalent co-morbidities were renal, cardiovascular and ophthalmic ones. Most patients had “ischemic DFU” and DFU in their “right” limb. The most prevalent location of DFU was patients’ toes, with most of them being in the big toe. 28.2% of the patients underwent lower-limb amputations. The amputation rate in the hospital where the “multidisciplinary approach” has been used was lower (23.7% vs. 30.1%).

**Conclusions:**

Number of patients with DFU is increasing. DFU is most likely to develop in middle-aged diabetic patients with a long duration of DM and poor blood sugar control who have other co-morbidities of DM. Male patients are at more risk. Recurrence of DFU is a major point of concern which underscores the importance of patient education to prevent secondary ulcers. As a result, educating medical and nursing personnel, applying screening and prevention guidelines, and allocating more resources are of great importance regarding treatment of DFU patients. Application of the “multidisciplinary approach” can reduce the rate of amputations. Primary care physicians might be furnished with the information presented in the present study.

## Background

One of the most important and disabling complications of diabetes mellitus (DM) is the diabetic foot ulcers (DFU). Development of DFU is traditionally believed to result from a combination of oxygen deficiency caused by peripheral vascular disease, peripheral neuropathy, minor foot traumas, foot deformities, and infection [1-3]. DFU, with a lifetime development risk of 15% [4], incidence of 1–4%, and prevalence of 5.3% to 10.5% [5,6] in all diabetic patients, accounts for more than half of the non-traumatic lower-extremity amputations in the world [7-11]. Globally, one lower limb is lost every 30 seconds because of DFU [12]. The range of mortality following diabetic foot amputation is 39–80% after 5 years, which is worse than the mortality rate for most malignancies [5]. Approximately, 20% of hospital admissions among diabetic patients are in consequence of foot problems [13]. Furthermore, DFU is among the most prevalent causes of hospitalization and morbidity [10,14] and is responsible for more days of hospital stay than any other chronic complication of DM [15,16].

According to estimation of the burden of DM based on Disability-Adjusted Life Years (DALYS) by the Endocrinology and Metabolism Research Center of Tehran University of Medical Sciences in Iran in 2001 [17], nearly 15% of overall burden of DM was due to foot neuropathy, DFU, and amputation. DFU lesions are significant health and socioeconomic problem as they exert adverse effects on patients’ quality of life and impose heavy economic burden on the patient and the state due to rising the need for rehabilitative and home care services [18,19]. Given the DFU’s high prevalence, heavy burden, and severe impact on patients’ life quality, it is advisable that sufficient heed be paid to prevention of this particular complication of DM. Furthermore, while effective treatment and formulation of prevention guidelines require a thorough understanding of characteristics of DFU patients and their ulcers, there are reports that not only patients’ but also physicians’ information about these characteristics is inadequate and even the process leading to ulceration and amputation is still not well understood by many healthcare professionals [20]. Indeed, several authors have reported the relative infrequency of foot evaluation by primary care physicians and surgeons; as in the primary care setting, only 23–49% of persons with DM have their feet evaluated on a yearly basis [21].

We conducted this descriptive study to investigate the characteristics of diabetic foot patients and their foot ulcers.

## Methods

This descriptive, retrospective study was carried out by including all patients admitted because of DFU between the years 2002 and 2008 in two tertiary university hospitals (Dr. Shariati Hospital and Imam Khomeini Hospital) in Tehran, capital of Iran. Medical archives of the patients were utilized and necessary data was collected using a predesigned data collection sheet. The information was thereafter entered into SPSS software, version 15, for analysis. The data collected comprised information on the patient’s age and sex, family history of DM, duration of DM and DFU, method of DM control, history of lower-limb amputation, history of previous hospitalizations, location of the foot ulcer, type of the foot ulcer, side of the foot ulcer (right or left limb), co-morbidities found during admission, laboratory data, duration of hospitalization, and the outcome.

We defined renal co-morbidity as presence of micro-albuminuria, macro-albuminuria, or end-stage renal disease; cardiovascular co-morbidity as presence of hypertension or ischemic heart disease; ophthalmic co-morbidity as presence of simple or proliferative diabetic retinopathy or cataract. We considered sensorimotor neuropathy in case of paresthesia, loss or reduction of vibration, pressure, temperature or superficial pain sensation or two-point discrimination (pin prick test), decreased sensation on screening with a monofilament (10-g), absence or reinforcement of Achilles tendon reflex or presence of deformities in the foot. We also defined autonomic neuropathy as presence of unexplained orthostatic hypotension, gastroparesis, dyspepsia, diabetic diarrhea or constipation, neurogenic bladder, erectile dysfunction, vaginal or skin dryness.

The study was reviewed and approved by the Ethics Committee of the Endocrinology and Metabolism Research Center of Tehran University of Medical Sciences. It conforms to the provisions of the Declaration of Helsinki.

## Results

**Figure 1:**
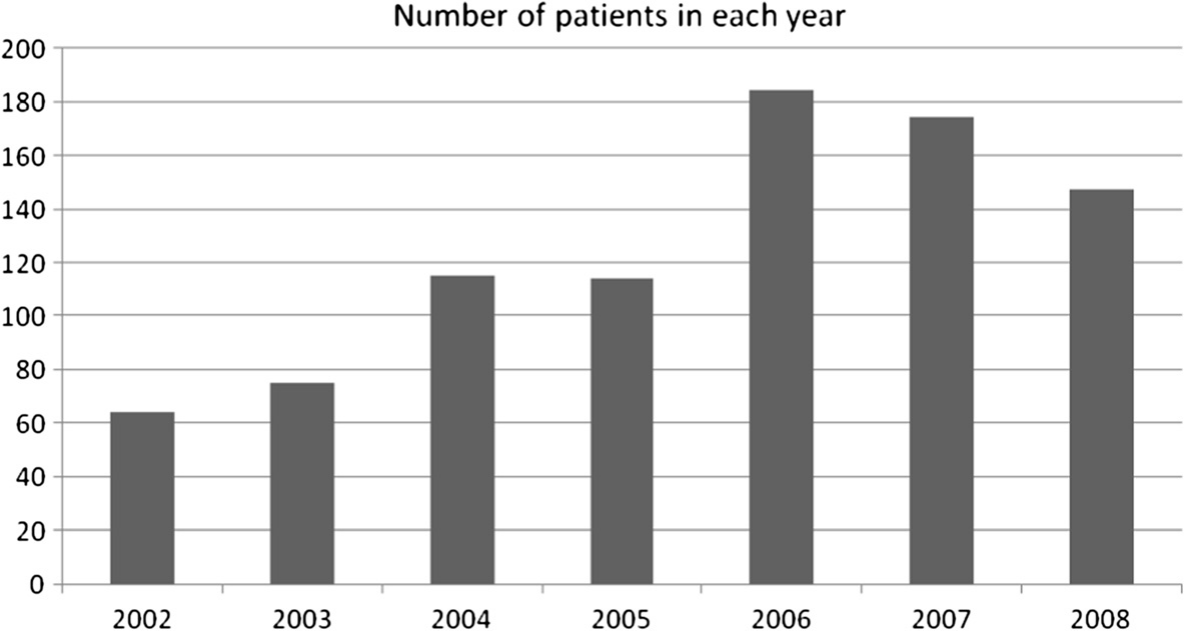
**Number of patients in each year.** This figure shows that total number of diabetic foot patients who were admitted in the studied hospitals during recent years is significantly more than that during the preceding years.

Medical archives of 873 patients were included. The number of the patients admitted in each year is illustrated in Figure 1. The basic characteristic data of the patients is shown in Table 1.

**Table 1:** Basic demographic characteristics of the patients

**Variable**	**Result**
Total number of patients	873
Male (%)	510 (58.1)
Female (%)	363 (41.9)
Mean age ± SD (year)	59.3 ± 11.2
Age (range in year)	25–87
Mean duration of DM ± SD (month)	172.2 ± 100.4
Duration of DM (range in month)	0–600
Positive family history of DM (%)	251 (28.8)

Only 4.18% of the patients had DM duration of 12 months or less. Mean duration of DFU prior to admission was 79.8 days. A total of 384 (43.99%) patients were under treatment with oral hypoglycemic agents, 397 (45.47%) were receiving insulin, 20 (2.29%) were receiving both oral hypoglycemic agents and insulin, and 72 (8.25%) were under no medication for blood sugar. Mean Hemoglobin A1C (HbA1C) level was 9.51%, and only 14.4% of the patients had HbA1C < 7%. Mean patients’ first fasting blood sugar during admission was 198.7 mg/dl. 69.6% of the patients had history of previous hospitalization due to DM complications. The causes of previous hospitalizations are shown in Table 2.

**Table 2:** Causes of patients’ previous hospitalizations (% of the patients)

**Complication**	**Percentage**
Previous DFU	22.4
Cardiovascular	9.8
Ophthalmic	7.9
Uncontrolled blood sugar	3
Cerebrovascular accident	2.8
Renal	2.4
Two or more of these complications	21.3

16.4% of the patients had previous lower-limb amputations, including major lower-limb amputation (above ankle) in 4% and minor lower-limb amputation (below ankle) in 12.4% of the patients.

During hospitalization, patients were evaluated about presence of DM co-morbidities. Data about presence of DM co-morbidities in the patients is shown in Table 3.

**Table 3:** Prevalence of DM co-morbidities in patients (% of the patients)

**Co-morbidity**	**Percentage**
Renal	60.3
Cardiovascular	59.1
Retinopathy	40.9
Sensorimotor neuropathy	27.5
Autonomic neuropathy	9

Renal co-morbidity: Micro-albuminuria, macro-albuminuria, or end-stage renal disease.

Cardiovascular co-morbidity: hypertension or ischemic heart disease.

Ophthalmic co-morbidity: simple or proliferative diabetic retinopathy, cataract.

74.1% of the patients had ischemic DFU, 17.4% had neuropathic DFU, and 8.5% had neuro-ischemic DFU. 53.4% of the patients had DFU in the right lower limb, 38.8% in the left lower limb, and 7.8% in both lower limbs.

Locations of DFUs in our patients are summarized in Table 4. The most prevalent location of DFU was patients’ toes, with most of them being in the big toe followed by the second toe. Twenty-six patients had DFU at the site of previous surgeries like amputation, debridement, or venous graft removal for coronary artery bypass graft (CABG).

**Table 4:** Locations of DFU (% of the patients)

**Location of DFU**	**Percentage**
Toe	44.5
Dorsum of foot	14.2
Plantar aspect	10.9
Heel	4.6
Shank	4.5
Site of previous surgery	2.9
Malleoles	2.7
Between toes	1.9
Lateral or medial aspects of foot	1.3
Ankle	1
More than one location	11.3

DFU: Diabetic foot ulcer.

Eventually 28.2% of the patients had undergone lower-limb amputations: 61.7% of the cases were minor amputations and 38.3% were major amputations.

Mean duration of hospitalization was 16.7 ± 11.3 days (range = 1–87 days), and the mortality rate was 5.2%.

## Discussion

Globally, the total number of people with DM is expected to rise from 171 million in 2000 to 366 million in 2030 [22]. Consistent with global predictions, total number of people with DM in Iran and accordingly, the number of patients with DFU are expected to increase. Figure 1 demonstrates that the overall number of DFU patients in our hospitals has risen in recent years. Similarly, the number of DFU patients admitted in these two hospitals had increased between the years 1979 and 2001 [23]. As a result, educating medical and nursing personnel, applying screening and prevention guidelines, and allocating more resources are of great importance in treatment of DFU patients.

Most of our patients developed DFU in 5th and 6th decades of their life, with the mean age being 59.3 years. Other studies also have found the average age of developing DFU to be about 55–60 years [23-26]. Male patients accounted for 58.1% of our total study population. Other investigators have also reported male patients to comprise 50–63.3% of their study populations [23-26]; and there is one study [27] reporting female patients to constitute the majority of the patients.

The high percentage (28.8%) of positive family history of DM in our patients underlines the important role of genetics in this condition. Mean duration of DFU was 79.8 days, which means that there was a two-month gap between DFU development and referral. Early treatment of DFU is an important factor in achieving better therapeutic results; consequently, it is vitally important that patients and primary care physicians be sufficiently educated and the former be encouraged to seek treatment earlier.

While in some patients (3 cases) DM was firstly presented with DFU, mean duration of DM in our patients was 172.2 months (14.3 years). In other similar studies in Iran, mean duration of DM was also about 14 to 15 years [23,26]. A study from Singapore [24] reported that duration of DM in 58.4% of the patients was more than 10 years. Only 4.18% of our patients had DM durations of 12 months or less which suggests that although DFU can develop in a diabetic patient at any time, it could be deemed a long-term complication of DM in most cases.

91.75% of our patients were under treatment to decrease their blood sugar; be that as it may, most of the cases (85.6%) had poor DM control (as indicated by their HbA1C levels > 7%) and 69.6% of the cases had preceding episodes of hospitalization due to DM co-morbidities. Chiming in with previous reports, these findings suggest that DFU is most likely to develop in diabetic patients with poor control of blood sugar [24,26,28,29]. Based on the fact that more than 50% of the patients had cardiovascular and renal and more than 40% had ophthalmic co-morbidities, we think that conducting a complete review of systems in DFU patients with emphasis on these systems is inevitable. Prevalence of sensorimotor and especially autonomic neuropathy was lower than similar studies. Considering the fact that diagnosis of autonomic neuropathy is based on history taking, the low prevalence of autonomic neuropathy could be in part due to incomplete history taking in some cases.

Several studies have conclusively shown that DFU is more common in patients with previous history of foot ulceration or amputation [30,31]. Apelqvist and colleagues found a DFU amputation recurrence rate of 34% after 1 year and 70% after 5 years [32]. Risk of DFU development in patients with history of previous ulcer is 57 times more than that of patients without this history [33]. Of our cases, 22.4% had history of previous hospitalization because of DFU and 16.3% had preceding lower-limb amputations. Therefore, recurrence of DFU is a major point of concern in DM patients, which underscores the importance of DFU prevention in DM patients and appropriate patient education to prevent secondary ulcers. Additionally, 47.2% of the cases had history of previous hospitalizations due to other co-morbidities of DM, including cardiovascular, cerebrovascular, renal, and ophthalmic complications. Furthermore, even a larger number of our patients had these co-morbidities based on evaluations made during their admission. Reports about the relationship between these co-morbidities and DFU development are controversial but many reports have concluded that presence of every single one of these co-morbidities is an independent risk factor for development of DFU and amputation [26,29,30,32].

We also found that most DFU cases were of the ischemic (74.1%) followed by the neuropathic type. Shojaiefard and colleagues [26] reported the same results and along with Nather and colleagues [24] concluded that presence of ischemic ulcer and gangrene was a risk factor for amputation. The ratio of right limb DFU to left limb DFU was 1:1.37. It seems that it is because most of the patients are right-dominant and use their right limb more frequently which might expose the right lower limb to more frequent traumas.

The most prevalent locations involved were toes (big toe followed by the second toe), plantar aspect of the foot, and dorsum of the foot, which accounted for more than 70% of the cases. Other investigators have reported similar results [25]. It seems that risk of DFU development in more distal parts of the limbs, which are prone to ischemia, diabetic neuropathy, and traumas, is much more than that of the proximal parts. Moreover, 26 patients developed DFU at the site of previous surgeries like amputation, debridement, or venous graft removal for CABG. Physicians should remember this point and avoid unnecessary surgical manipulations in lower limbs of DM patients.

Our patients were hospitalized for an average of 16.7 days; and at the end, 28.2% needed amputations. The minor-to-major amputation ratio was 1:1.61. The rate of amputation varies in different reports from different countries. The global rate is reported to be about 14–24% of the patients [34,35] and 15% in western countries [36]. The rate was 27.2% in a study in Singapore in 2008 [22] and 28.5% in Sudan in 2005 [25]. In Iran also there are different reports ranging from 40% in 1995 and 20% in 1999 [23] to 28.1% in 2005 [26]. An interesting result was the point that the amputation rate in one hospital was lower than that in the other (23.7% vs. 30.1%). At the hospital with the lower rate of amputation, since the year 2000 a “multidisciplinary approach “ has been adhered to for prevention and early case detection through patient and personnel education and multiple therapies (frequent debridement, drainage, washing, and dressing along with antibiotic therapy and daily assessment of the healing process) managed by a team of orthopedic surgeon, vascular surgeon, infectious diseases specialist, internist, endocrinologist, interventional cardiologist, nurse, general practitioner, and physiotherapist [23,26]. It is likely that application of this approach reduced the rate of amputation in DFU patients of this hospital. There are other reports about decreased rates of amputation thanks to this approach [23,37,38].

## Conclusions

Long duration of hospitalization of our patients in tandem with high percentage of the amputations and overall mortality rates highlights the high burden of DFU and the significance of its prevention and early treatment. Besides, number of our DFU patients is increasing which denotes the need for allocating more resources.

The status of DM care in our patients is far from satisfactory. Moreover, regarding the fact that DFU is most likely to develop in patients with poor DM control, it is inevitable that patient education should be afforded its due attention. In addition, while cardiovascular, renal and ophthalmic co-morbidities are common in DFU patients, it is important to conduct a complete review of systems (and not only approach to DFU) in DFU patients with emphasis on these systems. Primary care physicians should be furnished with the information presented in the present study. To conclude, a multidisciplinary approach can confer better treatment and outcome with respect to DFU.

## Abbreviations

DFU: Diabetic foot ulcer; DM: Diabetes mellitus; SD: Standard deviation; HbA1C: Hemoglobin A1C; CABG: Coronary artery bypass graft.

## Competing interests

The authors declare that they have no competing interests.

## Authors’ contributions

NM contributed to conception and designation of the study, acquisition, analysis and interpretation of data and drafted the manuscript. OTM contributed to conception and design and supervised data gathering. MRMT was the coordinator and revised the manuscript critically for important intellectual content. MP helped in coordination and gave final approval of the version to be published. RH and BL conceived of the study and participated in its design and coordination. All authors read and approved the final manuscript.
